# Bacterial Cellular Engineering by Genome Editing and Gene Silencing

**DOI:** 10.3390/ijms15022773

**Published:** 2014-02-18

**Authors:** Nobutaka Nakashima, Kentaro Miyazaki

**Affiliations:** 1 Bioproduction Research Institute, National Institute of Advanced Industrial Sciences and Technology (AIST), 2-17-2-1 Tsukisamu-Higashi, Toyohira-ku, Sapporo 062-8517, Japan; E-Mail: n-nakashima@aist.go.jp; 2 Biomass Refinery Research Center, National Institute of Advanced Industrial Sciences and Technology (AIST), 3-11-32, Kagamiyama, Higashi-Hiroshima, Hiroshima 739-0046, Japan; 3 Department of Medical Genome Sciences, Graduate School of Frontier Sciences, the University of Tokyo, 2-17-2-1 Tsukisamu-higashi, Toyohira-ku, Sapporo, Hokkaido 062-8517, Japan

**Keywords:** gene knockout, allelic exchange, gene knock-in, genome editing, RNA-guided nucleases, mobile group II intron, antisense RNA, gene knockdown, gene silencing

## Abstract

Genome editing is an important technology for bacterial cellular engineering, which is commonly conducted by homologous recombination-based procedures, including gene knockout (disruption), knock-in (insertion), and allelic exchange. In addition, some new recombination-independent approaches have emerged that utilize catalytic RNAs, artificial nucleases, nucleic acid analogs, and peptide nucleic acids. Apart from these methods, which directly modify the genomic structure, an alternative approach is to conditionally modify the gene expression profile at the posttranscriptional level without altering the genomes. This is performed by expressing antisense RNAs to knock down (silence) target mRNAs *in vivo*. This review describes the features and recent advances on methods used in genomic engineering and silencing technologies that are advantageously used for bacterial cellular engineering.

## 1. Introduction

Microorganisms have been used since ancient times for the production of fermented food products, such as cheese, sourdough, beer, wine, and vinegar, and humans have enjoyed the benefits of this biotechnology in terms of transforming raw materials to value-added products with superior flavor, texture, and longevity. Microorganisms can be found in almost every natural environment on our planet [1,2]. The traditional method for obtaining microorganisms that are useful for human life is to search for such microorganisms in the natural environment. Once the strain has been identified, it is cultured in the laboratory, and the original strain is often bred for higher performance through a randomized process. Subculturing has been employed to select for better performing progeny strains arising during cultivation. This “forward” genetics approach has been widely used in biotechnological processes for the production of, e.g., enzymes, foods, amino acids, and fine chemicals.

In recent years, an opposite “reverse” genetics approach has emerged. In this approach, genetic perturbations (mutations) are introduced into a particular gene of interest, and its impact is investigated through functional analysis [3]. Concomitant with the rapid accumulation of available genetic information, this reverse genetics approach is increasingly used for strain improvement in this synthetic biology age [4]. Specific genes residing in bacterial genomes (or chromosomal DNA) are targeted by various mutations, including knockout (disruption), knock-in (insertion), and allelic exchange [5]. All of these genomic modifications can be carried out following a similar experimental technique based on homologous recombination.

In addition to methods that directly (or permanently) modify genomic sequences, a novel approach has emerged that does not alter the genomic sequences, but rather alters the gene expression profile through conditionally repressing expression of mRNA at the translation level. This gene silencing or gene knockdown is carried out by antisense RNAs (asRNAs). The advantage of this silencing technology is its wide applicability, especially for genes essential to growth and thus not ready for disruption. In this review, we describe the principles, procedures, and important points for the above-mentioned methods from the perspective of bacterial cellular engineering.

## 2. Conventional Genome Editing

### 2.1. Gene Knockout

**Figure 1.**
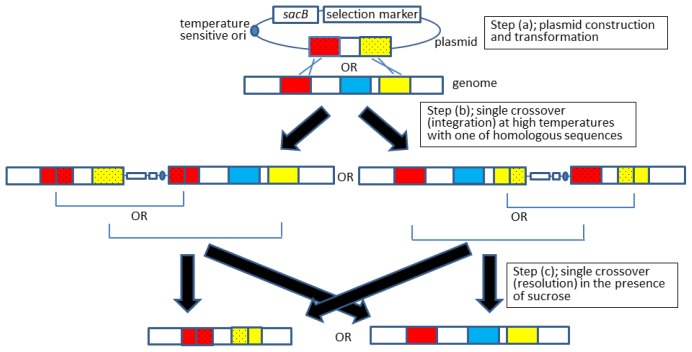
Schematic drawing of a gene knockout using the pSC101^ts^-*sacB* method [9]. Colored boxes denote open reading frames and blue boxes indicate the gene to be knocked out. The open reading frames that are derived from a plasmid are dotted. As homologous sequences on plasmids, fragments >500 base pairs are typically used. In this drawing, the first recombination (integration) occurs within the red (**middle left**) or yellow boxes (**middle right**). The second recombination (resolution) occurs using homologous regions within the genome, causing deletion (**bottom left**) or reconstruction to original organization (**bottom right**).

In 1989, a plasmid-based gene knockout method was developed in *Escherichia coli* that is achieved through two recombination events (*i.e*., integration and resolution) [6]. A plasmid carrying a replacing gene fragment containing homologous ends is integrated into a target gene locus through homologous recombination. To this end, the replacing gene fragment is cloned into a plasmid containing a temperature-sensitive replication origin, pSC101^ts^, which replicates at low temperatures (e.g., 30 °C), but not high temperatures (e.g., 43 °C ; Step (a) in Figure 1). Using the plasmid, *E. coli* is transformed and colonies are selected at a permissive, low temperature in the presence of the appropriate antibiotic. Transformants are then transferred to fresh medium and grown at a high temperature to prohibit self-replication of the plasmid. By selecting transformants in the presence of the antibiotic, the modified genomic fragment harbored on the plasmid is forced to integrate into the targeted genome locus through homologous recombination (Step (b) in Figure 1). After integration, the second recombination event takes place; resolved (disintegrated) progeny is selected at low, permissive temperatures at which the plasmid is able to replicate. During the resolving step, the specific region between the homologous sequences is deleted from the genome (Step (c) in Figure 1). This approach has been successfully applied to replace the *bolA* gene in the *E. coli* genome. However, the frequency to obtain the desired clone (“hit-rate”) was rather low and thus labor-intensive, especially at the resolution step [6]. It was hence improved by adding a counterselective marker gene onto the vector. The marker was a sucrose-sensitive suicide *sacB* gene of *Bacillus subtilis*; recombinants carrying the gene cannot grow on sucrose-containing plates, which are effective for the enrichment of clones lacking the gene during resolution. This improved method has been applied to various bacteria, including *E. coli* [6–9], *Myxococcus xanthus* [10], *Corynebacterium glutamicum* [11], *Rhodococcus* spp. [12], and *Pseudomonas putida* [13].

In 2000, Datsenko and colleagues developed a recombination-dependent, but slightly different, method for deletion of a genomic segment from *E. coli* using linear DNAs and λ-red recombinase [14]. Since then, this method has been applied to various bacteria including *Salmonella* spp. [15], *Mycobacterium tuberculosis* [16], *Streptomyces* spp. [17], and *B. subtilis* [18]. In brief, cells in which Gam, Bet, and Exo proteins of λ phage are expressed are transformed with linear DNAs containing homologous DNA sequences, two FRT sequences and a selection marker [14]. Then, the selection marker is removed by expressing the FLP recombinase that causes recombination between two FRT sequences [14].

Features of two representative gene knockout methods above are summarized in Table 1. The key feature of both methods is that no selection marker is left on the genomes, allowing multiple rounds of knockout. Note, however, that in the case of the original λ-red recombinase method by Datsenko and colleagues, a “scar” sequence of 81–85 base pairs (bp) in length is retained in the genome. When multiple rounds of disruption are performed, a risk exists of recombination between the scar sequences [19]. Furthermore, the scar sequence limits the precision of possible genomic modifications, which is easy with the pSC101^ts^-*sacB* method. The λ-red recombinase method has been widespread, but the pSC101^ts^-*sacB* method is still preferred for its several advantages, including the sequence-specificity and easiness for allelic exchange and knock-in, as described in Sections 2.2 and 2.3. A combination of these two methods has also been reported [20], and other variations are also known [7,21–23].

### 2.2. Allelic Exchange

**Figure 2.**
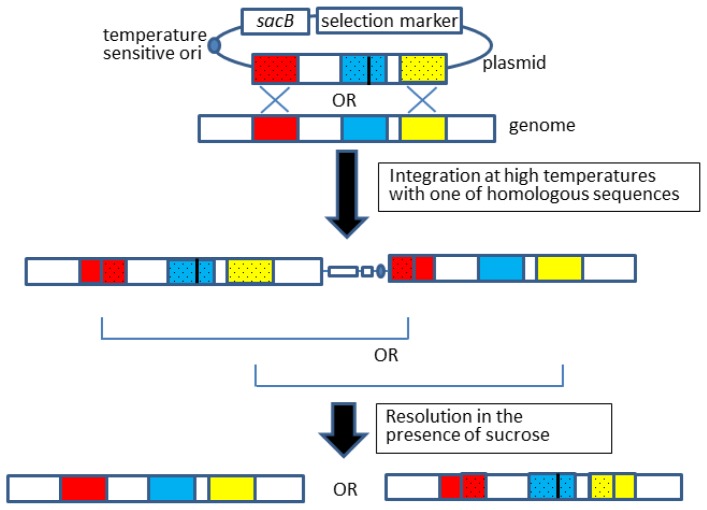
Allelic exchange. Colored boxes denote open reading frames and a black line in blue boxes indicates a point mutation to be introduced. The open reading frames that are derived from a plasmid are dotted. The integration step should occur via one of the two homologous regions, but only one of the two integration patterns is shown here for simplicity.

Allelic exchange is a modified version of gene knockout that can be carried out by following the same procedure for gene knockout (Section 2.1), which is illustrated in Figure 2 [8,9].

**Figure 3.**
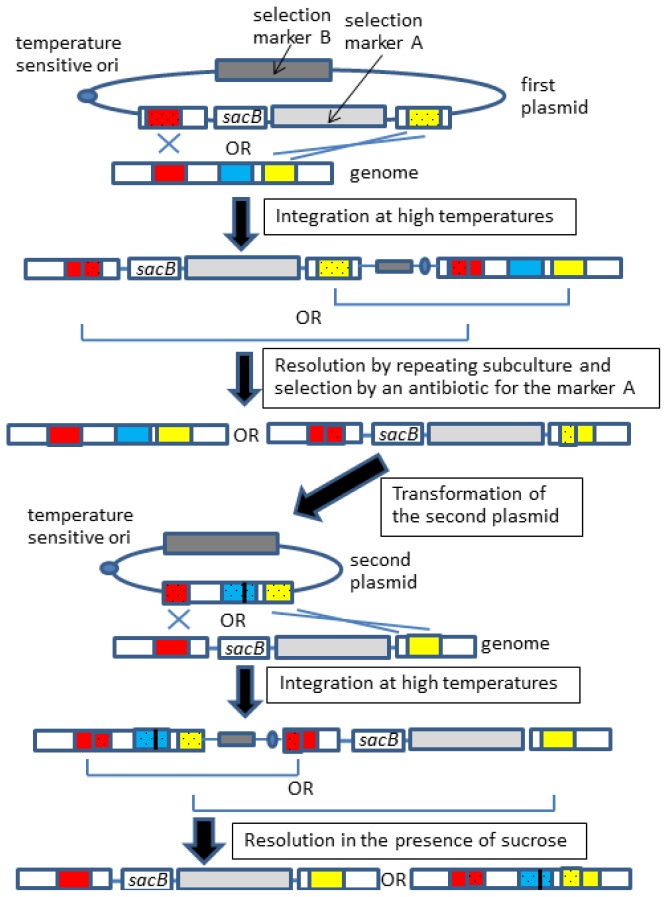
Improved method for allelic exchange. Light and dark gray boxes indicate different selection marker genes. Other boxes and lines are the same as in Figure 2. The open reading frames that are derived from a plasmid are dotted. The integration step should occur via one of the two homologous regions, but only one of the two integration patterns is shown here for simplicity.

Overall, the gene knockout and allelic exchange procedures are quite similar; however, the latter requires an observable phenotypic change because confirmation of fragment exchange is not readily achievable by simple methods, such as colony polymerase chain reaction (PCR), but instead requires DNA sequencing. Furthermore, by this method, we failed to introduce an mlc* allele, which contained a point mutation in the *mlc* gene of *E. coli* [24]. This was probably due to the low rate of proper resolution to obtain desired recombinants, which was a common problem in the gene knockout experiments as described above. To overcome these problems, Emmerson and colleagues applied two mechanisms for the resolution step: antibiotic selection and sucrose-based counterselection (Figure 3) [7], which was originally conducted by single sucrose-counterselection. Although Emmerson’s method requires an additional plasmid and, thus, is rather labor-intensive, this modification is effective for recovering the desired allelic exchange of various genes (e.g., *mlc* [24], *crp* [24], and *lee4* [9]).

Another allelic exchange method is getting popular, which is mediated with single-stranded oligonucleotides and the λ-red recombinase [25]. For this method, only a Beta protein of three λ-red proteins is required, and the Beta protein binds single-stranded DNAs and promotes annealing to the homologous DNAs [25]. The important feature is high efficiency, allowing a recombination without any selection. In one report, 25% of the *E. coli* cell population was successfully recombined in the absence of antibiotic marker selection and any selection pressure [25]. Furthermore, because no plasmid constructions are required, multiple-rounds of allelic exchange are easily achieved. However, there is a serious limitation for usage in *E. coli*; the mismatch repair system of host cells should be removed for high efficiency, because mismatched nucleotides always occur at the initial step of recombination. Therefore, occurrence rate of undesirable mutations is increased [25]. To circumvent this disadvantage, it is reported that usage of modified (unnatural) bases at the mismatched sites are effective [26], and other improvements for usability are also reported [27,28].

In bacteria other than *E. coli*, this method has been used in *M. tuberculosis* [29], *Pseudomonas syringae* [30], *Lactobacillus reuteri* [31], and *Lactococcus lactis* [31].

### 2.3. Gene Knock-in

**Figure 4.**
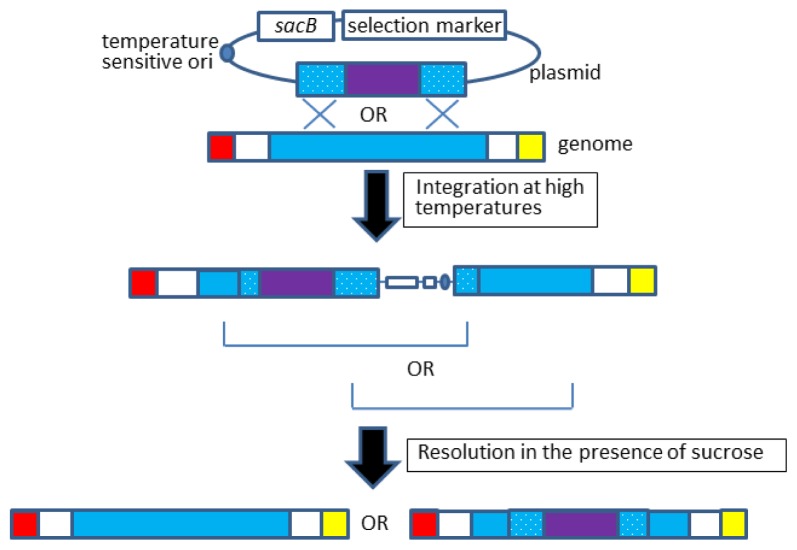
Gene knock-in. Blue box indicates a gene locus to which a target gene is integrated. Red and yellow boxes are neighboring genes, and purple boxes indicate the heterologous gene to be knocked-in. The open reading frames that are derived from a plasmid are dotted. The integration step should occur via one of the two homologous regions, but only one of the two integration patterns is shown here for simplicity.

The gene knock-in procedure is very similar to that of gene knockout and allelic exchange [32–34], as outlined in Figure 4. For example, our group knocked-in a “doxycycline inducible promoter–T7 RNA polymerase gene” cassette into the *lacZ* locus of *E. coli* [32]; the *lacZ* locus was chosen because it has no or little effect on cell growth. When the resulting strain was transformed with plasmids having a “T7 promoter-gene of interest” cassette, it was successful in expressing the gene of interest in a doxycycline-dependent manner [32].

Gene knock-in is most advantageously employed when the use of plasmid vectors is inadequate or when the copy number of the gene of interest should be kept low. Note also that knocked-in genes are more stably inherited to progenies compared to genes on plasmids [35].

## 3. New Technologies for Genome Editing

### 3.1. Gene Knockout with Mobile Group II Introns

In 2001, a novel method to knockout bacterial genes was reported, which uses mobile group II introns [36]. Group II introns are naturally occurring genetic elements found in eubacteria, mitochondria, and plastids [37–39]. *L. lactis* Ll.LtrB is the most studied group II intron, which is a ribonucleoprotein (RNP) consisting of an intron RNA and an LtrA protein [40,41]. The intron RNA excises itself from the RNA transcript through a lariat structure and also encodes LtrA protein, a reverse transcriptase. Ll.LtrB selects a DNA site to integrate into the intron RNA through both protein–DNA interactions and RNA–DNA base pairings [42–44]. Redesigning the intron RNA in Ll.LtrB allows any gene to be targeted [43]. When host cells are transformed with a vector expressing a redesigned intron RNA and intact LtrA, the redesigned Ll.LtrB recognizes the target DNA sequence and integrates itself (both intron RNA and LtrA protein participate in target-site recognition). Then, LtrA reverse transcribes the integrated sequence, and the DNA repair system of host cells repairs the target site, completing gene knockout.

Computer-aided design is carried out for Ll.LtrB intron RNAs [43,45] via the Sigma-Aldrich TargeTron Design Site for purchasers of the TargeTron Gene Knockout System (http://www.sigmaaldrich.com/life-science/functional-genomics-and-rnai/targetron.html) [43] and the Targetronics web site (http://www.targetrons.com/). Additionally, it is available to the research community free of charge at http://clostron.com [46].

Advantages of this method compared to conventional methods are its high efficiency, high specificity (low ectopic integration rate), and applicability to a broad range of bacteria. It has been applied to knockout genes in *E. coli* [36,47], *Shigella flexneri* [36], *Salmonella enterica* [36], *L. lactis* [48], *Clostridium* spp. [46,49,50], *Staphylococcus* spp. [51–53], *Pseudomonas* spp. [47,54], and *Agrobacterium tumefaciens* [47,54], *Azospirillum brasiliense* [55], *Francisella tularensis* [56], *Listeria monocytogenes* [57], *Paenibacillus alvei* [58], *Pasteurella multocida* [59], *Ralstonia eutropha* [60], *Yersinia pseudotuberculosis* [61,62], *Sodalis glossinidius* [63], and *Bacillus anthracis* [64]. This method is most extensively applied for the modification of *Clostridium* spp. genomes as artificial homologous recombination events are difficult to cause in this genus [46]. In addition, a thermophilic targetron system has been reported in *Clostridium thermocellum* [49]. In *E. coli*, a library of randomized target site recognition sequences has been constructed and used to introduce insertion mutation throughout the genome [65].

In 2013, an improved method was reported in which mobile group II introns and Cre/*lox* recombination system was combined (GETR, Genome Editing via Targetrons and Recombinases) [66]. The method was developed for applicability in broad bacterial host range and usage in gene knockout, knock-in, and other large-scale genome modifications. At least, the genomes of *E. coli*, *S. aureus*, *B. subtilis*, and *Shewanella oneidensis* are amenable to this method [66].

### 3.2. RNA Guided-, Artificial Endonuclease Mediated-, and Peptide Nucleic Acid Stimulated-Recombination

Very recently, a novel mechanism was discovered in *Streptococcus* spp.; two small RNAs interacted with a Cas9 endonuclease and guided the enzyme to a specific DNA sequence through DNA–RNA hybridization [67,68]. Next, the targeted DNA underwent blunt-ended and double-stranded breakage by Cas9. This mechanism is called clustered regularly interspaced short palindromic repeats (CRISPR)/CRISPR-associated (Cas) systems. Bacteria possess this mechanism to protect themselves from potentially toxic intruders, such as viruses and plasmids. The small RNAs can be redesigned to guide Cas9 to virtually any DNA sequence, and this finding have paved a possibility of a new method for genome editing in both eukaryotes and prokaryotes [67,69–73]. In 2013, the CRISPR-Cas system has been applied for allelic exchange in *Streptococcus pneumoniae* and *E. coli* with high efficiency [74]; 100% and 65% of resulting colonies had expected mutations in *S. pneumoniae* and *E. coli*, respectively. This high efficiency is due to cytotoxic nature of the CRISPR-Cas gene cassette that has targeted to genomic loci, and cells having the mutated target loci can escape from the cytotoxicity [75]. However, we would like to emphasize that construction and design of complex plasmids are necessary as a disadvantage of the method.

In eukaryotes, genome editing using zinc finger nucleases (ZFNs) or transcription activator-like effector nucleases (TALENs) is becoming increasingly popular [75–77]. TALENs are artificial endonucleases created by fusing DNA-binding domain of transcription activator-like effector protein (secreted by *Xanthomonas* spp.) and the DNA cleavage domain of the FokI restriction enzyme [78]. Importantly, these nucleases can easily be engineered to cleave any DNA sequence [79,80]. Cleaved DNAs are repaired by nonhomologous end-joining, which is stimulated by double-stranded breaks [81], causing base replacement or deletion. This method can be combined with homologous recombination to cause gene knock-in by introducing exogenous DNA fragments in parallel [80,82].

Peptide nucleic acids (PNAs) are synthetic nucleotides. Oligomeric PNAs can hybridize to DNAs or RNAs as natural oligonucleotides [83–85]. Unlike natural nucleic acid oligomers that are connected by phosphodiester bonds, PNA oligomers are connected by peptide bonds and thus resistant to both nucleases and proteases. Furthermore, PNAs form more stable PNA–DNA and PNA–RNA duplexes than DNA–DNA and DNA–RNA duplexes because PNAs do not have a negative charge on their backbone, and electrostatic repulsion between complementary strands is absent. PNAs can also form PNA–DNA–PNA triplexes [86]. Studies have shown that PNA–DNA–PNA triplexes at specific genomic sites can stimulate the DNA repair machinery and homologous recombination in mammalian cells [87]. Indeed, successful knockout has been reported at the *CCR5* gene locus [88].

### 3.3. Possibility of the Brand-New Methods for the Future

Several new methods for genome editing are described in the above two sections, but these methods have not been applied to bacteria widely. Efficiency of genome editing in bacteria is summarized and listed in Table 2. In the case where the artificial nuclease method was applied to knock-out genes in murine embryonic stem cells, 8% of cells had the disrupted gene in maximum [89]. This high efficiency allowed identifying disrupted cells without using any selection marker. All the conventional methods in bacteria require selection markers, as occurrence frequency of correct homologous recombination is usually low (Table 2). Therefore, the new methods are expected to become popular if a method without selection markers would be established. Some bacteria have too low homologous recombination frequencies to apply conventional methods [90], and genomes of such bacteria may be manipulated only by the new methods. The other important point is reducing off-target knockouts that are confirmed for artificial nucleases [91]. In some bacteria, expression vectors and transformation procedures have not been established yet and plasmid-less methods should be developed. We further would like to point out that nuclease-based methods involve double stranded DNA brakes that are difficult to be repaired in bacteria and may cause problem of cytotoxicity [74].

## 4. Gene Silencing Using asRNAs

### 4.1. asRNAs Expressed from Expression Vectors

One can change the nature of bacteria without editing the genome. One method is to silencing target mRNAs by expressing asRNAs [93–99]. This method was first reported in 1984 in *E. coli* [100]. The largest advantage of using asRNAs is the conditionality of the silencing effect, making it possible to apply to genes essential for growth. Creating expression vectors for asRNAs is less laborious than gene knockout methods.

**Figure 5.**
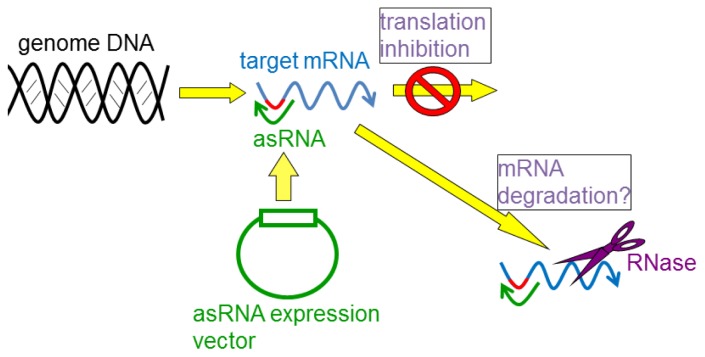
Mechanism of asRNAs. Red lines indicate a ribosome-binding site (RBS) on the target mRNA.

In many cases, asRNAs are designed to hybridize to the ribosome-binding site (RBS) and the start codon region of the target mRNAs (Figure 5) [98]. This is because translation initiation is the limiting step in the translational process; thus, preventing the ribosome from binding to the RBS site of target mRNAs is most critical for its efficacy. However, the factors that are most affected by knocking down the targeted mRNAs is still unclear, although some mechanisms have been proposed. In *E. coli*, the level of target mRNAs decrease following silencing as well as protein level [93–95,101,102]. It is, thus, likely that target mRNAs that are masked with asRNAs tend to be rapidly degraded in the cell. Probably, asRNA-targeted mRNAs are free from ribosomes (so-called naked mRNAs) and easily accessible to the nucleases compared to mRNAs in the polysome states [103].

**Figure 6.**
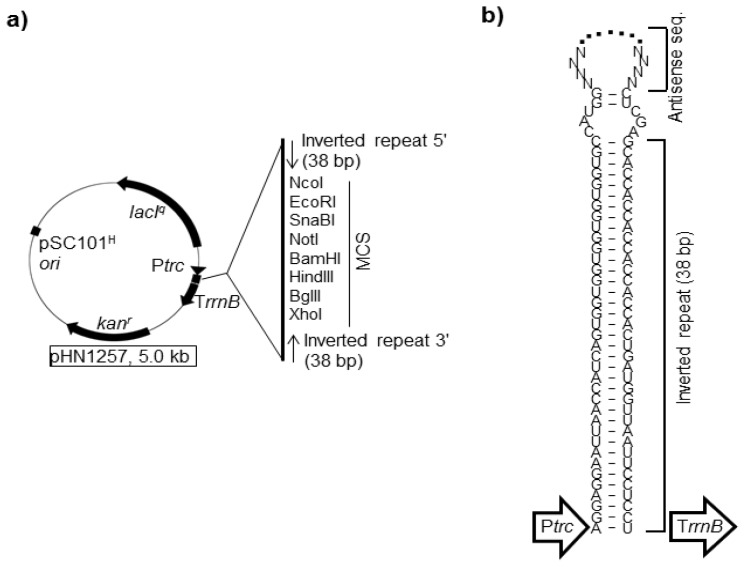
Schematic map of the HPasRNA expression vector, pHN1270. (**a**) Arrows indicate open reading frames (*lacI^q^* and kanamycin-resistance gene, *kan^r^*), the *trc* promoter (P*trc*), or *rrnB* terminator (T*rrnB*), and a box indicates the pSC101^H^ replication origin (high copy version of pSC101). Restriction enzyme sites in the multiple cloning site (MCS) are unique; (**b**) The structure of HPasRNAs is shown. “NNNN….NNNN” indicates an antisense sequence or a MCS control sequence in the case of an empty plasmid.

Until recently, the asRNA-mediated gene silencing method was disadvantageous, especially in *E. coli*; the silencing efficacy varied greatly depending on the targeted gene and was generally low [104]. We and other groups attempted to increase the efficacy by redesigning the expression system of asRNAs. In 2006, we found that asRNAs combined by a hairpin structure (hairpin asRNAs; HPasRNAs), had much higher silencing efficacies than those lacking the hairpin structure in *E. coli* (Figure 6) [95]. The hairpin structure improves stability of the asRNAs and extending the lifetime in cells. Indeed, for several genes (e.g., *fabI* and *ackA*) [94], expected phenotypes did not appear upon expression of asRNAs lacking the hairpin structure, but clearly appeared upon expression of HPasRNAs. The HPasRNA expression plasmid (pHN1257) contained the *trc* promoter (P*trc*) and the lactose repressor gene (*lacI^q^*), which drive conditional expression of HPasRNAs with IPTG. When DNA fragments containing the reverse complements of RBS and start codon sequences of target genes are cloned under the control of the P*trc* in the multiple cloning site (MCS) of pHN1257, HPasRNAs containing antisense sequences at the loop region are expressed. Antisense sequences of 80–150 nucleotides in length are sufficient for specific hybridization of mRNAs-HPasRNAs [95]. Furthermore, an additional three plasmids harboring different selection markers and replication origins were constructed [94]. These four plasmids, including pHN1257, were co-transformable (compatible) in any combination and were used to silencing up to four genes simultaneously [94]. We confirmed that four genes were silenced simultaneously, and the silencing efficacy of each gene was comparable to those of their respective single silencing [94].

To observe a distinct phenotype by asRNA-mediated gene silencing, the key parameter is the amount of the cellular asRNA level over target mRNAs [95,103]. In bacteria, transcription and translation take place simultaneously in the same location [103]. For successful gene silencing, the expressed asRNA must bind the target mRNA before the ribosome. As the ribosome is the most abundant molecule in cells, one must maximize the expression of asRNAs by using strong transcriptional promoters. In our studies, a strong P*trc* was used, which showed improved results compared to weaker promoters, such as arabinose-inducible and tetracycline-inducible promoters [32,95]. The vector copy number also affects the efficiency, and using a higher copy number plasmid gave better results [94]. Detailed procedures for constructing fine silencing plasmids have been described earlier [93]. Expression vectors for asRNAs developed by us have been distributed to many researchers. Table 3 summarizes the selected applications using the vectors.

The asRNA-mediated gene silencing approach has been proven to be effective not only in *E. coli* but also in various other bacteria, including *S. aureus* [108], *Clostridium* spp. [109], *Bacillus megaterium* [110], *Streptomyces* spp. [111], *Lactobacillus rhamnosus* [112], and *Mycobacterium* spp. [113,114]. In one outstanding report in 2006, a genome-wide shotgun library of asRNAs was constructed by cloning genomic fragments of *S. aureus* downstream of the tetracycline-inducible promoter [108]. This systematic approach led to the identification of growth essential genes, which can be a promising target for new antibiotics. Presently, all bacteria are thought to have naturally occurring small RNAs that act as asRNAs [115], and therefore, this method should be applicable to other bacteria.

Recently, two studies have been published where the asRNA-mediated gene silencing has been applied for rationally designing metabolic pathway of *E. coli* [116,117]. In both reports, over 70 genes were silenced to screen gene targets that increase productivity of valuable compounds. Such large scale screens are feasible with this method but not with gene knock out, because any *E. coli* strain is used and multiple silencing is easy.

### 4.2. Antisnese Oligonucleotides Synthesized in Vitro

Antisense oligonucleotides (both DNAs and RNAs) that are synthesized *in vitro* are convenient to silence target RNAs, because they can be added directly to bacterial cultures whenever desired, without constructing plasmids [118]. Therefore, synthetic asRNAs are suitable for bacteria for which expression vectors have not been developed. Stability of antisense oligonucleotides in bacterial cells (in other words, tolerance to nuclease) can be improved by incorporating unnatural modified nucleotides, such as Locked Nucleic Acids [119] or phosphoroathioate oligonucleotides [120]. As PNAs (see Section 3.2) have high stability in cells and high target site specificity than natural oligonucleotides, they also work as effective antisense silencers [83,84].

**Figure 7.**
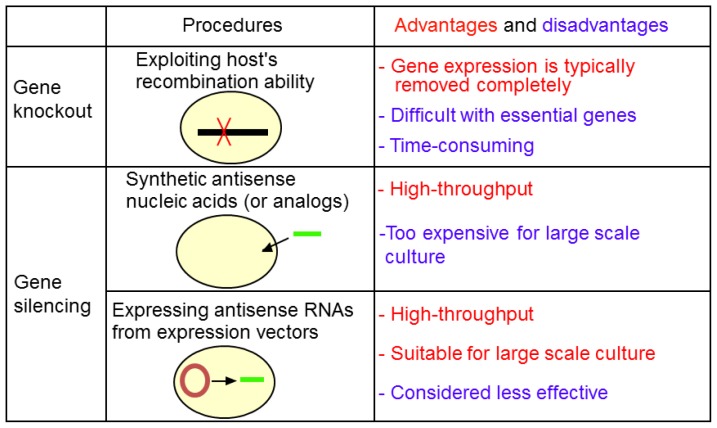
Summary of gene knockout and gene silencing.

However, synthetic asRNAs are costly and hardly fits to large-scale cultures compared to expressed asRNAs. Permeability of synthetic asRNAs across bacterial cell membranes should be taken into account. When PNAs are applied to *E. coli*, permeability of PNAs should be increased by attaching “cell penetrating peptides” to PNAs [83,84]. Figure 7 summarizes features of expressed and synthetic antisense methods as well as gene knockout method.

## 5. Conclusions

Here, we described the methods for genome editing and gene silencing, including conventional and new ones. The research trend of this area is correspondence to high-throughput and large-scale analyses. To this end, high recombination efficiency and selection-free approaches (e.g., not using antibiotic markers) are required for genome editing. Gene silencing with expressed asRNAs well suites to high-throughput analyses and indeed come into usage [116,117]. Once the asRNA expression libraries that cover whole bacterial genome are established, then everyone can use the libraries as valuable research resources almost permanently. In addition to the above points, the methods that can be used in many bacteria universally are required for the future.

We believe that the further development of genome editing and gene silencing methods are necessary for understanding cellular functions as a system and for altering metabolic functions as desired.

## Figures and Tables

**Table 1.** Comparison of two gene knockout methods a.

Compared points	pSC101^ts^-*sacB* method by Blomfield *et al*. [8]	λ-Red recombinase method by Datsenko *et al*. [14]
Recombination via	Two times of single crossover	One time of double crossover and FLP (flippase)–FRT recombination
Enzymes for recombination	endogenous enzymes	λ Gam, Bet, Exo, and flippase
Reliability	Low due to resolution of original gene organizations	**High**
Host requirements	Only recombination-proficient hosts	**Any**
Plasmid construction	Necessary	**unnecessary**
Transformation efficiency required	**Low**	High
Transformation procedures required	**Once**	Twice
Marker gene used for integration	**Not retained**	**Not retained**
Unnecessary genome arrangement	**No**	Yes, leaving an 81–85-bp “scar” sequence [19]
Bacteria proven to be applicable	*E. coli* [6–9], *M. xanthus* [10], *C. glutamicum* [11], *Rhodococcus* spp. [12], and *P. putida* [13]	*E. coli* [14], *Salmonella* spp. [15], *M. tuberculosis* [16], *Streptomyces* spp. [17], and *B. subtilis* [18]

a Advantageous features are shown in bold.

**Table 2.** Comparison of typical efficiency of genome editing.

Host bacteria	Method used	Efficiency	Reference
*E. coli*	λ-red recombinase method, double stranded DNA	10^3^ to 10^4^ recombinants per 10^8^ viable cells a	[92]
*E. coli*	λ-red recombinase method, single stranded DNA	~10^7^ recombinants per 10^8^ viable cells	[92]
*E. coli*	λ-red recombinase method, single stranded DNA	25% b	[25]
*L. reuteri*	λ-red recombinase method, single stranded DNA	0.4%–19% b	[31]
*E. coli*	mobile group II introns	1%–80% b	[43]
*C. thermocellum*	mobile group II introns	67%–100% b	[49]
*S. aureus*	mobile group II introns	37%–100% b	[51]
*S. pneumoniae*	CRISPR-Cas9 system	100% b	[74]
*E. coli*	CRISPR-Cas9 system	65% b	[74]

a Replacing the *galK* gene with a drug cassette;

b Efficiency is calculated as percentage of successful recombination per appeared colonies without any selection pressure.

**Table 3.** Genes silenced with HPasRNAs in *E. coli*.

Gene name	Gene product	Silencing efficacy a	Observed phenotypes upon expression of HPasRNAs
*lacZ*	β-galactosidase	88% [93]	–
*ackA*	Acetate kinase	78% [94]	Reduced acetate production, no growth on minimal acetate media [95]
*aceE*	Pyruvate dehydrogenase component		Acetate auxotroph, accumulation of pyruvate [93]
*ftsZ*	Tubulin-like protein		Severe growth (essential gene), elongated cell [105]
*fusA*	Elongation factor G		Severe growth (essential gene), sensitization 12-fold to fusidic acid [106]
(Many growth essential genes)			Construction of a shotgun genomic library expressing HPasRNAs, identification of growth essential genes [106]
*mutT*	Protein for maintaining DNA replication fidelity	>90% [106]	Protein level control in a stepwise fashion by changing concentration of expression inducer (IPTG) [107]
*mutS*, *mutD*, *ndk* (triple silencing)	Proteins for maintaining DNA replication fidelity		Increased mutation rate by 2000-fold over wild-type cells [94]

a Evaluated as reduced protein activity upon HPasRNA expression.
